# Effective silencing of miR-126 after ischemic stroke by means of intravenous α-tocopherol–conjugated heteroduplex oligonucleotide in mice

**DOI:** 10.1038/s41598-021-93666-y

**Published:** 2021-07-09

**Authors:** Motohiro Suzuki, Satoru Ishibashi, Eri Iwasawa, Takahiro Oguma, Yasuhiro Saito, Fuying Li, Shinichi Otsu, Keiko Ichinose, Kotaro Yoshioka, Tetsuya Nagata, Takanori Yokota

**Affiliations:** 1 Department of Neurology and Neurological Science, Tokyo Medical and Dental University, 1-5-45 Yushima, Bunkyo-ku, Tokyo, 113-8519 Japan; 2 Department of Internal Medicine, Fukaya Red Cross Hospital, Saitama, Japan; 3 Mitsubishi Tanabe Pharma Corporation, Osaka, Japan

**Keywords:** Molecular biology, Neuroscience, Neurology

## Abstract

Brain endothelial cells (BECs) are involved in the pathogenesis of ischemic stroke. Recently, several microRNAs (miRNAs) in BECs were reported to regulate the endothelial function in ischemic brain. Therefore, modulation of miRNAs in BECs by a therapeutic oligonucleotide to inhibit miRNA (antimiR) could be a useful strategy for treating ischemic stroke. However, few attempts have been made to achieve this strategy via systemic route due to lack of efficient delivery-method toward BECs. Here, we have developed a new technology for delivering an antimiR into BECs and silencing miRNAs in BECs, using a mouse ischemic stroke model. We designed a heteroduplex oligonucleotide, comprising an antimiR against miRNA-126 (miR-126) known as the endothelial-specific miRNA and its complementary RNA, conjugated to α-tocopherol as a delivery ligand (Toc-HDO targeting miR-126). Intravenous administration of Toc-HDO targeting miR-126 remarkably suppressed miR-126 expression in ischemic brain of the model mice. In addition, we showed that Toc-HDO targeting miR-126 was delivered into BECs more efficiently than the parent antimiR in ischemic brain, and that it was delivered more effectively in ischemic brain than non-ischemic brain of this model mice. Our study highlights the potential of this technology as a new clinical therapeutic option for ischemic stroke.

## Introduction

Ischemic stroke is a leading cause of death worldwide. The pathophysiology of ischemic stroke is currently understood through the conceptual framework of the neurovascular unit (NVU), which includes brain endothelial cells (BECs), astrocytes, pericytes, and neurons^1,2^. Cellular interplay between all components of the NVU influence functional recovery following ischemic stroke^1^. At the level of the NVU, BECs play a central role in the pathogenesis of ischemic stroke^3–5^.

MicroRNAs (miRNAs) are small non-coding RNAs^6,7^. MiRNAs bind to the 3′-untranslated region (UTR) of their target mRNAs, inhibiting their translation and destabilizing them^6,7^. Many miRNAs have tissue-specific expression patterns and a cell type–specific function^6^. Indeed, several miRNAs in BECs affect endothelial function in ischemic stroke^8–13^. Therefore, modulation of miRNAs in BECs at the molecular level could be an effective therapeutic strategy for ischemic stroke.

AntimiRs are antisense oligonucleotides (ASOs) that inhibit miRNA function by directly binding to miRNAs^14^. However, when administered in the absence of a carrier, antimiRs achieve limited tissue distribution^6^. Thus, an improved delivery system is required for therapeutic applications of antimiRs, such as for treatment of ischemic stroke.

Recently, we developed a novel system whereby a heteroduplex oligonucleotide (HDO), comprising an ASO as the parent strand and its complementary RNA, is conjugated to α-tocopherol (Toc) as a delivery ligand^15,16^. Compared with the corresponding ASO, intravenously administered Toc-conjugated HDOs (Toc-HDOs) were more effectively delivered into BECs of normal mice where they more efficiently downregulated the expression of a BEC-specific target mRNA^15^. However, it is unclear whether miRNA-targeting Toc-HDOs with an antimiR as the parent strand can be delivered into BECs and regulate miRNA in BECs under conditions of ischemic stroke.

In this study, to investigate the effect of this novel platform technology in BECs, we selected miRNA-126-3p (miR-126) as the target miRNA because miR-126 is specifically expressed in endothelial cells^17–19^ and its endothelium-specific properties are well-studied^20,21^.

We report that, following intravenous administration of a Toc-HDO targeting miR-126, its delivery to ischemic brain tissue was remarkably higher than that of the corresponding single-stranded antimiR, and that it substantially inhibited the function of miR-126 under conditions of ischemic stroke.

## Results

### Selection of the most effective antimiR sequence

**Figure 1:**
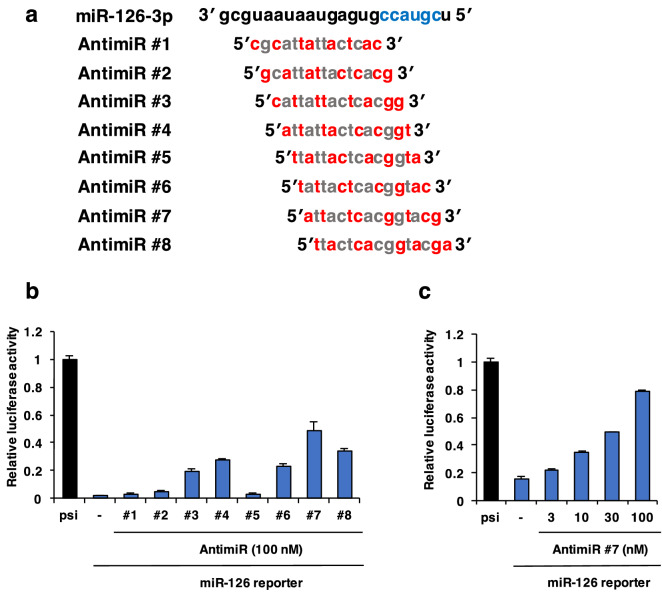
Selection of the most effective antimiR sequence for targeting microRNA-126 (miR-126). (**a**) Schematic representation of the candidate antimiRs targeting miR-126. Blue, seed sequence; red, locked nucleic acids (LNAs); gray, DNAs (**b**, **c**) Efficiency of the candidate antimiRs measured by luciferase reporter assay. (**b**) The indicated antimiRs at 100 nM or (**c**) antimiR #7 at 3, 10, 30, and 100 nM were co-transfected with psiCHECK-2 reporter plasmid into bEnd3 cells. Relative luciferase activity was measured at 24 h after transfection. Abbreviations: miR-126 reporter, psiCHECK-2 reporter plasmid that contains the binding site for miR-126 in the 3′ UTR of the *Renilla* luciferase gene; psi, psiCHECK-2 empty vector that does not contain the binding site for miR-126. Data are presented as the mean ± s.e.m. (n = 3 per group).

**Figure 2:**
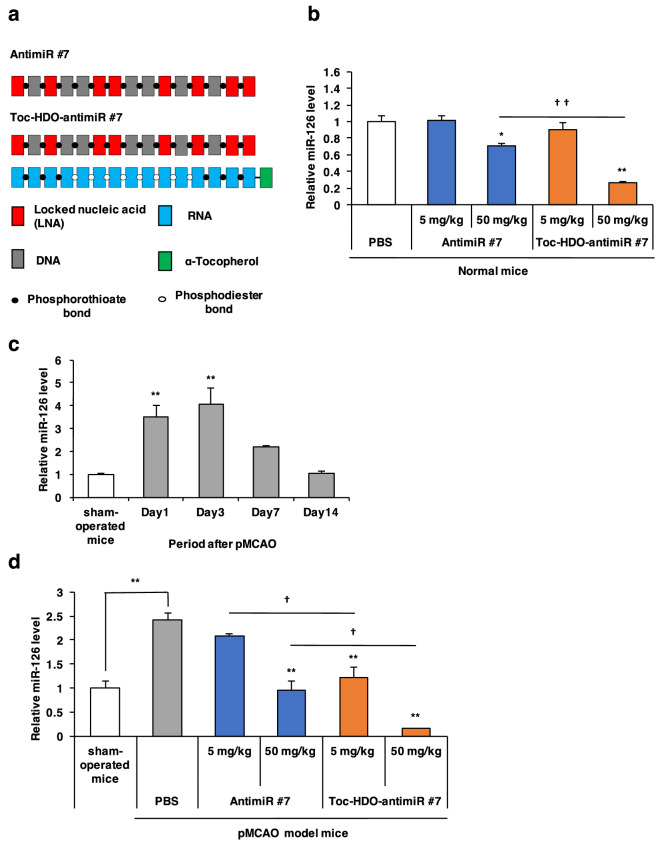
Inhibition of microRNA in BECs after intravenous administration of Toc-HDO-antimiR #7. (**a**) Design of Toc-HDO-antimiR #7 and antimiR #7. (**b**) Quantitative reverse transcription PCR (qRT-PCR) analysis of miR-126 expression in the cerebral cortex from normal mice after intravenous administration of phosphate buffered saline (PBS), antimiR #7, or Toc-HDO-antimiR #7 at 5 mg/kg or 50 mg/kg (*P < 0.05, **P < 0.01 versus PBS control; ††*P* < 0.01 versus antimiR #7). (**c**) Time course of miR-126 expression in the ischemic region after permanent middle cerebral artery occlusion (pMCAO) (**P < 0.01 versus sham-operated mice). (**d**) qRT-PCR analysis of miR-126 expression in the cerebral cortex from sham-operated mice and the ischemic region from pMCAO model mice after intravenous administration of PBS, antimiR #7, or Toc-HDO-antimiR #7 at 5 mg/kg or 50 mg/kg (**P < 0.01 versus PBS control; †*P* < 0.01 versus antimiR #7). All data are relative to U6 small RNA levels and are presented as the mean ± s.e.m (n = 3 per group). *P* values were calculated using one-way ANOVA followed by (**c**) Dunnett’s test or (**b**, **d**) Tukey–Kramer test.

To select the most effective antimiR sequence, we designed a series of antimiRs targeting miR-126. Chemical modification of oligonucleotides, such as phosphorothioate (PS) backbone modification and sugar modifications including locked nucleic acids (LNAs), can enhance nuclease resistance and binding affinity towards RNAs and proteins^6,14,22,23^. The antimiRs designed here were 15-mer LNA/DNA mixmer-type and fully PS-modified antisense oligonucleotides. These antimiRs sequences ranged from sequences that did not bind to the seed region in the miR-126 sequence, specificity determinant for miR-126 target recognition, to sequences that contains the full-length reverse complement of the seed region. The chemical modifications of the antimiRs (Figs. 1a, 2a) were the same as those of miravirsen (SPC3649), which is one of the most advanced antimiRs^14,24^. To evaluate the efficiency of the candidate antimiRs in a luciferase reporter assay, we used a psiCHECK-2 reporter plasmid harboring the complementary sequences of miR-126 in the 3′ UTR of the *Renilla* luciferase gene (miR-126 reporter). As a control, we used psiCHECK-2 empty plasmid without the complementary sequences of miR-126. These two reporter plasmids were co-transfected with the candidate antimiRs into cells of the mouse brain endothelial cell line, bEnd3, which endogenously expresses miR-126. In this experiment, antimiR #7 recovered the inhibition of luciferase activity by endogenous miR-126 in bEnd3 cells more effectively than other antimiRs (Fig. 1b), and in a dose-dependent manner (Fig. 1c). This result suggests that antimiR #7, which contains the full-length reverse complement of the seed region, bound to the target sequence most effectively among the candidate antimiRs in vitro. Therefore, we selected the antimiR #7 sequence for use as the parent strand of the Toc-HDO (Toc-HDO-antimiR #7) used in the experiments below. Also, PS modifications were introduced into the 3′- and 5′-wing portions of the complementary RNA in Toc-HDO-antimiR #7 (Fig. 2a).

### MiR-126 expression levels were downregulated by Toc-HDO-antimiR #7 in the cerebral cortex under normal condition

Normal mice were intravenously administered with phosphate buffered saline (PBS; vehicle control), antimiR #7, or Toc-HDO-antimiR #7 at doses corresponding to 5 mg/kg or 50 mg/kg of antimiR #7. The mice were euthanized 7 days later, and brain tissues from the cerebral cortex were isolated for quantitative reverse transcription PCR (qRT-PCR).

In normal cerebral cortex, qRT-PCR analysis showed that Toc-HDO-antimiR #7 at 50 mg/kg significantly lowered miR-126 expression (75% reduction,* P* < 0.001) compared with the PBS control. Toc-HDO-antimiR #7 at 50 mg/kg was significantly more effective in reducing miR-126 expression than antimiR #7 at the equivalent dose (*P* = 0.001). However, there was no significant difference in miR-126 expression between Toc-HDO-antimiR #7 and antimiR #7 treatment at 5 mg/kg (Fig. 2b).

To evaluate possible adverse effects of Toc-HDO-antimiR #7, we performed serum biochemical and histological analysis at 7 days after intravenous administration of Toc-HDO-antimiR #7. In normal mice, Toc-HDO-antimiR #7 at 5 mg/kg, 15 mg/kg, or 50 mg/kg did not elevate the level of aspartate transaminase, alanine transaminase, blood urea nitrogen, or creatinine in blood serum from normal mice (see Supplementary Fig. S1a online). Also, hematoxylin and eosin staining of kidney and liver sections revealed no histological abnormalities after administration of Toc-HDO-antimiR #7 at 50 mg/kg (see Supplementary Fig. S1b online and Supplementary Table S1 online). These examinations indicate that Toc-HDO-antimiR #7 did not induce renal or hepatic toxicity.

### Temporal profile of miR-126 expression in ischemic stroke

Using mice that had been subjected to a permanent middle cerebral artery occlusion (pMCAO), we evaluated the temporal profile of miR-126 expression after ischemic stroke. In these pMCAO model mice, infarction was distributed in the ipsilateral cerebral cortex, with almost no striatal involvement. The ischemic region was separately dissected from the infarction of the ipsilateral hemisphere, while the contralateral cortex was collected from the contralateral non-ischemic hemisphere. The expression level of miR-126 was quantified by qRT-PCR at 1, 3, 7, and 14 days after the pMCAO operation. The miR-126 expression in the ischemic region increased significantly at 1 and 3 days after pMCAO to reach approximately 3.5 and 4.1 folds (*P* = 0.004 and *P* = 0.001, respectively) than the sham-operated control levels (Fig. 2c). These data show that miR-126 expression was upregulated in the ischemic region following pMCAO.

### MiR-126 expression levels were downregulated by Toc-HDO-antimiR #7 in the ischemic brain region under ischemic stroke

pMCAO model mice at 3-h post operation were intravenously administered PBS, antimiR #7, or Toc-HDO-antimiR #7 at 5 mg/kg or 50 mg/kg. In the ischemic brain region of pMCAO model mice, miR-126 expression levels were significantly downregulated by Toc-HDO-antimiR #7 at 5 mg/kg (50% reduction,* P* < 0.001) and 50 mg/kg (94% reduction,* P* < 0.001) compared with the PBS control. In addition, Toc-HDO-antimiR #7 at 5 mg/kg and 50 mg/kg downregulated the miR-126 expression significantly more efficiently than antimiR #7 (*P* = 0.012 and *P* = 0.022, respectively) (Fig. 2d). These results showed that the intravenously administered Toc-HDO-antimiR #7 at 5 mg/kg downregulated the miR-126 expressions more effectively than antimiR #7 in the ischemic brain, but not in the cerebral cortex under normal condition, indicating that Toc-HDO-antimiR #7 be able to efficiently silence the target miRNA in BECs of ischemic brain.

### Delivery of Toc-HDO-antimiR #7 in ischemic stroke

**Figure 3:**
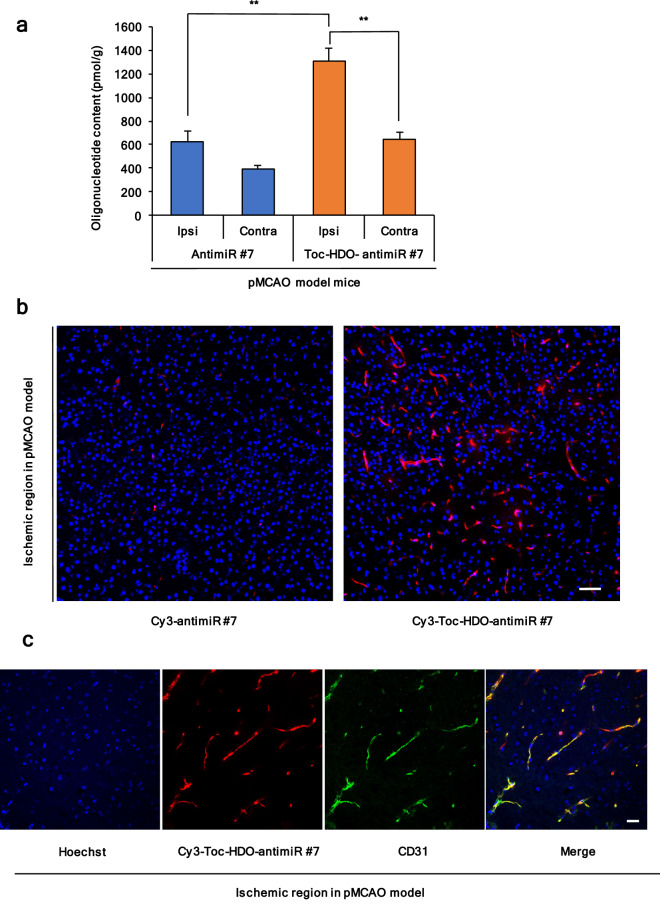
Delivery of Toc-HDO-antimiR #7 in ischemic stroke. (**a**) Oligonucleotide concentrations in the ischemic brain region of the ipsilateral hemisphere (ipsi) and the non-ischemic brain tissue of the contralateral hemisphere (contra) in pMCAO model mice after injection of Cy3-labeled Toc-HDO-antimiR #7 or Cy3-labeled antimiR #7. Data are presented as the mean ± s.e.m (n = 3 per group). **P < 0.01 versus the ipsi in pMCAO model mice after injection of Cy3-labeled Toc-HDO-antimiR #7 (one-way ANOVA followed by Tukey–Kramer test) (**b**) Confocal laser scanning microscopic images of brain sections in the ischemic region after injection of Cy3-labeled Toc-HDO-antimiR #7 or Cy3-labeled antimiR #7 (red). Scale bar, 50 µm. (**c**) Immunofluorescence images of Cy3-labeled Toc-HDO-antimiR #7 (red) and antibody against endothelial cell marker (anti-CD31; green), and Hoechst staining (blue). Scale bar, 25 µm.

We examined the delivery of Toc-HDO-antimiR #7 in the brain tissues following pMCAO. To enable quantification, Toc-HDO-antimiR #7 and antimiR #7 were tagged with Cy3 at the 5′ end of the antimiR sequence to produce Cy3-labeled Toc-HDO-antimiR #7 and Cy3-labeled antimiR #7, respectively. pMCAO model mice (3 h post-operation) were intravenously administered Cy3-labeled Toc-HDO-antimiR #7 or Cy3-labeled antimiR #7 at 15 mg/kg; the mice were euthanized 1 h later. By quantifying Cy3 signal intensities, we found that accumulation of Cy3-labeled Toc-HDO-antimiR #7 in the ischemic region of the ipsilateral hemisphere was 2.1 fold that of Cy3-labeled antimiR #7, and was 2.0 fold that of Cy3-labeled Toc-HDO-antimiR #7 in the non-ischemic brain tissue of the contralateral hemisphere (Fig. 3a). Both these differences were statistically significant (*P* < 0.001).

Secondly, to investigate the distribution of Toc-HDO-antimiR #7 following pMCAO, we evaluated brain slices by confocal laser scanning. In the ischemic region, Cy3-labeled Toc-HDO-antimiR #7 was highly accumulated along the brain microvasculature, which was observed as linear structures (Fig. 3b). Additionally, Cy3-labeled Toc-HDO-antimiR #7 co-localized with a BEC molecular marker, CD31, suggesting that Toc-HDO-antimiR #7 was successfully delivered into BECs (Fig. 3c). Conversely, Cy3-labeled antimiR #7 in the ischemic region and Cy3-labeled Toc-HDO-antimiR #7 in the contralateral cortex displayed weak signals following pMCAO (Fig. 3b, and see Supplementary Fig. S2a online). Furthermore, the sections were stained with an anti-PS antibody after intravenous administration of Cy3-labeled Toc-HDO-antimiR #7. The anti-PS antibody recognizes the backbone of the PS-containing Toc-HDO-antimiR #7^25^. Anti-PS staining showed robust signals of linear structures in the ischemic region as opposed to weak signals in the contralateral cortex (see Supplementary Fig. S2b online).

These results show that Toc-HDO-antimiR #7 was delivered into BECs more efficiently than antimiR #7 in ischemic stroke. In addition, Toc-HDO-antimiR #7 was delivered more efficiently in ischemic brain tissue than in non-ischemic brain tissue.

### Identification of downstream genes directly affected by miR-126 under the normal condition

To identify downstream genes that are directly suppressed by miR-126, we performed microarray analysis of (a) BECs isolated from normal mice and (b) bEnd3 cells. In the in vivo experiment, BECs were isolated by magnetic-activated cell sorting (MACS) from the brain tissues of normal mice treated with Toc-HDO-antimiR #7 at 5 mg/kg, 15 mg/kg, or 50 mg/kg. In the in vitro experiment, bEnd3 cells were transfected with antimiR #7 at 30 nM.

The candidate genes satisfied the following three criteria: (1) Gene expression was upregulated 1.5 fold or more in BECs isolated from the normal mice after injection of Toc-HDO-antimiR #7 at 50 mg/kg compared with PBS control; (2) Gene expression was upregulated 2.5 fold or more in bEnd3 cells transfected with antimiR #7 at 30 nM compared with non-treated control; and (3) The gene was registered in multiple miRNA target prediction programs, including miRTarBase, Target scan, and miRDB. On the basis of the microarray analysis, five genes (*Vcam1*, *VegfA*, *Akt2*, *Sgpl1*, and *Gbp2*) were identified as the candidate downstream genes of miR-126 (see Supplementary Table S2 online).

**Figure 4:**
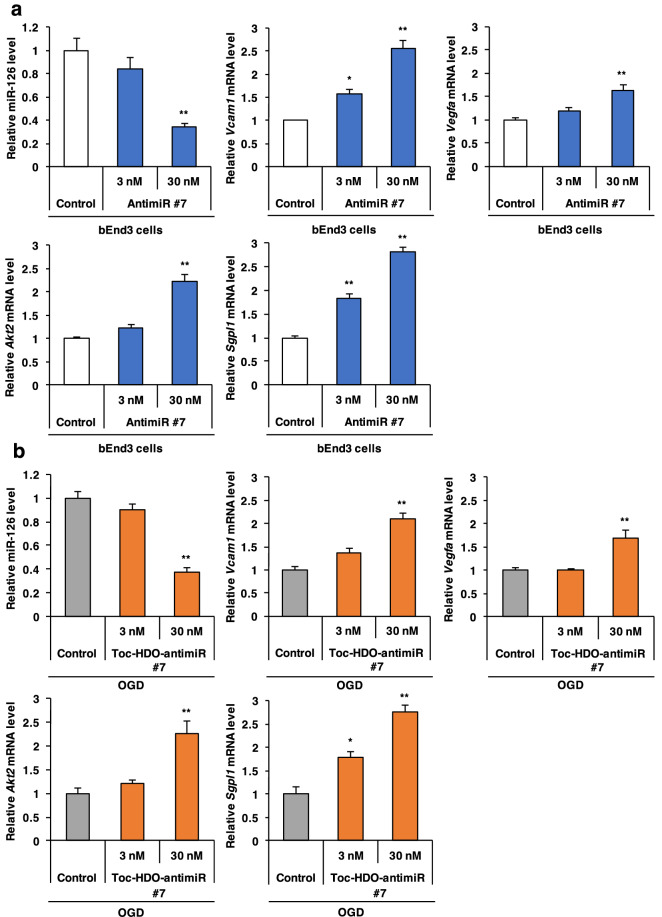
Upregulation of the miR-126 downstream genes by Toc-HDO-antimiR #7 *in vitro* under ischemia. (**a**) qRT-PCR analysis of the level of miR-126 and the mRNA levels of the candidate miR-126 downstream genes, *Vcam1*, *Vegfa*, *Akt2*, and *Sgpl1*, in bEnd3 cells transfected with antimiR #7 at 3 nM or 30 nM. (**b**) qRT-PCR analysis of the post- oxygen–glucose deprivation (OGD) level of miR-126 and mRNA levels of the miR-126 downstream genes in bEnd3 cells transfected with Toc-HDO-antimiR #7 at 3 nM or 30 nM. Expression levels are relative to U6 small RNA levels for miR-126 expression experiments and glyceraldehyde-3-phosphate dehydrogenase (*Gapdh*) mRNA levels for mRNA experiments. Data are presented as the mean ± s.e.m. (n = 3 per group; *P < 0.05, **P < 0.01 versus non-treated control). *P* values were calculated using one-way ANOVA followed by Dunnett’s test.

We performed qRT-PCR analysis to confirm that the candidate genes were directly suppressed by miR-126. When bEnd3 cells were transfected with antimiR #7 by using Lipofectamine 2000, antimiR #7 at 30 nM significantly reduced the miR-126 expression levels (63% reduction compared with non-treated control, *P* = 0.003) and upregulated the mRNA levels of four of the five candidate downstream genes, *Vcam1* (255% upregulation, *P* < 0.001), *Vegfa* (162% upregulation, *P* = 0.006), *Akt2* (223% upregulation, *P* < 0.001), and *Sgpl1* (281% upregulation, *P* < 0.001) in bEnd3 cells (Fig. 4a), but did not significantly upregulate *Gbp2* (data not shown).

To confirm that Toc-HDO-antimiR #7 and antimiR #7 bind to the target miRNA in a sequence-dependent manner, we measured the expression levels of miR-126 and the remaining four candidate genes in bEnd3 cells after transfection with Toc-HDO-antimiR scramble or antimiR scramble, in which the antimiR sequences are a scrambled sequence of miR-126. Neither Toc-HDO-antimiR scramble nor antimiR scramble reduced miR-126 expression levels and altered the expression levels of the candidate genes (see Supplementary Fig. S3 online). In addition, we confirmed that there is an miR-126 binding site within the 3′ UTR of each of the candidate genes (see Supplementary Fig. S4 online). Taken together, these data show that *Vcam1*, *Vegfa*, *Akt2*, and *Sgpl1* are direct downstream genes of miR-126.

### Upregulation of miR-126 downstream genes by Toc-HDO-antimiR #7 under ischemia in vitro

We evaluated whether Toc-HDO-antimiR #7 upregulates the expression of the miR-126 downstream genes under ischemia in bEnd3 cells in vitro. Oxygen–glucose deprivation (OGD) was utilized as an in vitro model of ischemic stroke^26^. The bEnd3 cells were subjected to OGD, transfected with Toc-HDO-antimiR #7 by using Lipofectamine 2000, and then analyzed 2 days later. Toc-HDO-antimiR #7 at 30 nM significantly upregulated the mRNA levels of the four miR-126 downstream genes: *Vcam1* (211% upregulation, *P* < 0.001), *Vegfa* (168% upregulation, *P* = 0.007), *Akt2* (227% upregulation, *P* = 0.003), and *Sgpl1* (275% upregulation, *P* < 0.001) (Fig. 4b).

### Upregulation of miR-126 downstream genes by Toc-HDO-antimiR #7 in ischemic stroke

**Figure 5:**
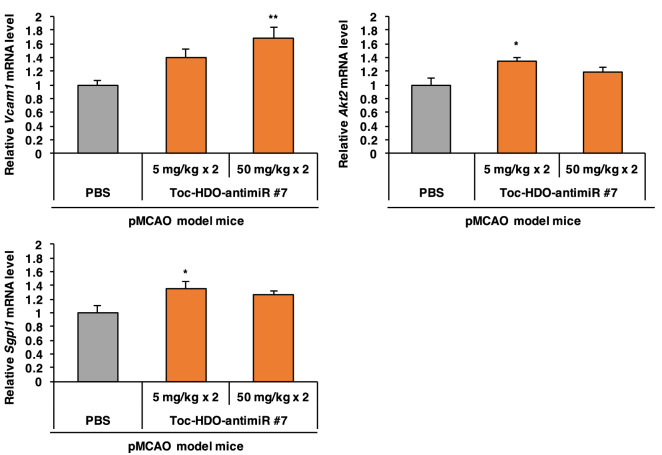
Upregulation of miR-126 target genes by Toc-HDO-antimiR #7 in ischemic stroke. qRT-PCR analysis of the mRNA levels of the miR-126 downstream genes, *Vcam1, Akt2*, and *Sgpl1* in the ischemic region of pMCAO model mice after two intravenous injections (at 1-week intervals) of Toc-HDO-antimiR #7 at 5 mg/kg or 50 mg/kg. Data are relative to *Gapdh* mRNA levels and are presented as the mean ± s.e.m. (n = 3 per group. *P < 0.05, **P < 0.01 versus PBS control). *P* values were calculated using one-way ANOVA followed by Dunnett’s test.

To assess the expression levels of miR-126 downstream genes in vivo, Toc-HDO-antimiR #7 at 5 mg/kg or 50 mg/kg was administered intravenously to mice at 3 h and 7 days after pMCAO; the mice were euthanized 2 weeks after pMCAO. Toc-HDO-antimiR #7 at 50 mg/kg significantly upregulated *Vcam1* (169% upregulation, *P* = 0.009) in the ischemic region. Toc-HDO-antimiR #7 at 5 mg/kg, but not 50 mg/kg, significantly upregulated *Akt2* (134% upregulation, *P* = 0.035) and *Sgpl1* (136% upregulation, *P* = 0.043) in the ischemic region (Fig. 5), but did not significantly upregulated *Vegfa* (data not shown). These data indicate that intravenously administered Toc-HDO-antimiR #7 has the potential to regulate the function of miR-126 in ischemic stroke.

We also examined whether Toc-HDO-antimiR #7 affected the brain damage after ischemic stroke. pMCAO model mice were intravenously injected with PBS, antimiR #7, or Toc-HDO-antimiR #7 at 50 mg/kg two times as described above. To assess neuronal damage, the brain sections were immunostained for the neuronal marker NeuN. Confocal microscopy analysis revealed the number of NeuN-positive neurons decreased in the peri-infarct region compared to the contralateral cortex. Furthermore, there were no differences in the number of NeuN-positive neurons at the peri-infarct region among the PBS group (mean ± s.e.m, 71 ± 5.5/mm^2^ of ROI), the antimiR #7 group (76 ± 4.5/mm^2^ of ROI), and the Toc-HDO-antimiR #7 group (74 ± 5.2/mm^2^ of ROI) (see Supplementary Fig. S5a online). In addition, we tested the effects of silencing miR-126 on infarct volume after pMCAO. The examination showed that there were no significant differences in infarct volume among the PBS group (mean ± s.e.m, 17% ± 1.1%), the antimiR #7 group (20% ± 1.4%), and the Toc-HDO-antimiR #7 group (21% ± 2.6%) at 14 days after pMCAO (see Supplementary Fig. S5b online). Thus, Toc-HDO-antimiR #7 was not found to affect the brain damage following pMCAO.

## Discussion

Here we developed a brand-new technology for delivering an antimiR to BECs in the ischemic region in a mouse model of stroke. In the pMCAO mouse model, intravenously administered Toc-HDO with the selected antimiR #7 as the parent strand more effectively reduced miR-126 expression levels in the ischemic region compared with the single-stranded antimiR #7. The accumulation of Toc-HDO-antimiR #7 was remarkably higher than that of the antimiR #7 in the ischemic region, and Toc-HDO-antimiR #7 was delivered successfully into BECs in the ischemic region. Additionally, the expression of miR-126 downstream genes, such as *Vcam1*, *Akt2*, and *Sgpl1*, were upregulated in the ischemic region after Toc-HDO-antimiR #7 treatment.

In ischemic stroke, the cerebral blood flow in the ischemic core, i.e., the central part of ischemic region, falls to a level 15%–20% lower than that in normal brain tissue, thereby reducing drug supply to the ischemic region^27,28^. However, despite the reduction in cerebral blood flow, intravenously administered Toc-HDO-antimiR #7 achieved remarkable delivery to BECs of the ischemic region. In our previous reports, we considered that Toc-HDO was delivered into the target cell at least in part by lipoprotein receptor–mediated uptake along the transport pathway of α-tocopherol after Toc-HDO bound to lipoproteins in mouse serum^15,16,29^. Lipoprotein receptors such as LDLR, LDLR-related protein 1 (LRP1), and scavenger receptor class B type 1 (SR-B1) are expressed on BECs^30–32^. The levels of these lipoprotein receptors are remarkably upregulated in BECs in the ischemic region in acute ischemic stroke^33,34^, suggesting that lipoprotein receptor–mediated endocytosis driven by ischemic stroke may enable effective delivery into BECs. Therefore, we presume that the higher accumulation of Toc-HDO-antimiR #7 than antimiR #7 in BECs of the ischemic region was due to upregulated lipoprotein receptors in these BECs. Also, this implies that Toc-HDO targeting the other miRNAs could be delivered into BECs more efficiently than the corresponding antimiRs in ischemic stroke, because Toc-HDO is considered to distribute along the physiological pathway of α-tocopherol^15,16^.

On the other hand, we consider that the enhanced in vivo silencing potency of the Toc-HDO-antimiR #7 was also associated with the intracellular silencing mechanism of an HDO, which is independent on the delivery ligand^15,16^. Our previous study demonstrated that an HDO-antimiR differed from its parent antimiR in the mechanism by which it silences the target miRNA^35^. The silencing mechanisms of antimiRs can be subdivided into two major categories: (1) steric-blocking type antimiRs sequester the target miRNA in an antimiR/target miRNA duplex, (2) degradation-type antimiRs induce degradation of the target miRNA^23,36,37^. AntimiRs with the same chemical modifications as the parent antimiR used in the present study competitively inhibit the target miRNA by steric-blocking, but do not induce target miRNA degradation^23,35,36^. In contrast, the intracellular silencing mechanism of an HDO-antimiR is both degradation of the targeted miRNA and steric-blocking^35^. Therefore, we presume that Toc-HDO-antimiR #7 inhibits the function of miR-126 in BECs by both mechanisms. However, further studies are required to establish the inhibitory mechanism of Toc-HDO-antimiR #7.

We previously showed that intravenous administration of a Toc-HDO targeting a single mRNA could efficiently silence the target mRNA in BECs under normal conditions^15^. However, treatment targeting a single mRNA by Toc-HDO may not be suitable for ischemic stroke pathophysiology, because it is mediated by multifaceted molecular mechanisms. In fact, inhibition of a single protein in BECs, e.g., inhibition of intracellular adhesion molecule-1 (ICAM-1) by a monoclonal antibody, has not been an effective treatment for ischemic stroke^38^. MiRNA-targeted therapy has the potential to regulate multiple mRNAs, because a single miRNA can alter multiple mRNAs^39^. Indeed, Toc-HDO antimiR #7 upregulated multiple mRNAs such as *Vcam1*, *Akt2* and *Sgpl1* mRNAs, which are suppressed directly by miR-126. Also, in acute ischemic stroke, reduced blood supply rapidly induces the biochemical cascade including failure of ionic gradients and excitotoxicity, leading to a cell death^39,40^. When an mRNA encodes a protein with a half-life long not enough to be reduced during the acute phase after ischemic stroke, silencing the mRNA as target molecule may not be therapeutically effective for the lack of the protein reduction. However, an advantage of silencing miRNA is that mRNA and protein expression can be upregulated regardless of the protein half-life. In the past, inhibition of miR-15a/16-1 is reported to maintain endothelial function such as BBB integrity by upregulating downstream mRNA and protein expressions in acute ischemic stroke, leading to improved functional outcome^12,41^. Hence, we anticipate that Toc-HDO targeting miRNAs will be an invaluable technology for the treatment of acute ischemic stroke.

In this study, we selected miR-126 as the target miRNA. This miRNA directly suppresses the expression of VCAM1 which is an intercellular adhesion molecule in endothelial cells^19,20^. In a previous report, overexpression of miR-126 through intracerebral injection of a lentivirus carrying the miR-126 gene reduced VCAM1 expression in brain tissues under ischemic stroke^42^. Here, we demonstrated that miR-126 knockdown by Toc-HDO-antimiR #7 resulted in upregulated *Vcam1* expression in the ischemic brain tissues. This implies that intravenously administered Toc-HDO-antimiR #7 successfully modified the functionality of miR-126 in ischemic stroke. In contrast, our examination revealed no difference in infarct volume in comparison with PBS after treatment with either antimiR #7 or Toc-HDO-antimiR #7. Consistent with this result, it has been reported that genetic deletion of endothelial miR-126 did not affect infarct volume in mice subjected to pMCAO, as was the case in our experiment^43^.

In the future, selection of novel effective miRNAs in BECs as therapeutic targets is essential for the development of Toc-HDO targeting miRNAs to improve functional prognosis following ischemic stroke.

This study has a limitation that although qRT-PCR was used to measure the miR-126 expression levels in brain tissues, qRT-PCR data does not always reflect the functional effects of an antimiR^23,44^. Therefore, we used luciferase reporter assay to confirm the miRNA silencing effects of the antimiRs in vitro, and we investigated the expression levels of miR-126 downstream genes in the ischemic region following Toc-HDO-antimiR #7 treatment.

In summary, our study demonstrates that intravenously administered Toc-HDO targeting miR-126 can amplify the delivery in BECs compared with the corresponding antimiR under ischemic stroke. We anticipate that Toc-HDO targeting miRNAs will advance new therapeutic strategies in ischemic stroke.

## Methods

### Animal experiments

Wild-type male BALB/cCrSlc mice at 6 to 8 weeks of age (Sankyo Laboratory Animal Center, Tokyo, Japan) were used in this study. All animal experiments were performed in accordance with the National Institutes of Health Guide for the Care and Use of Laboratory Animals and ARRIVE guidelines. This study was approved by the Institutional Animal Care and Use Committee of Tokyo Medical and Dental University (Approval number: A2020-084A).

### Permanent middle cerebral artery occlusion (pMCAO) model

As described previously^33,45^, mice were anesthetized using isoflurane gas, and an incision was made vertically in the skin at the midpoint between the left orbit and the external auditory canal. The left middle cerebral artery (MCA) was exposed through the skull after the temporal muscles were reflected. A small burr hole was made on the skull surface. The left MCA was coagulated with a microbipolar electrocoagulator (Summit Hill Laboratories, Tinton Falls, USA). The temporal muscle was replaced, and the skin was sutured. Mice were kept in a recovery cage until they recovered from anesthesia. Sham surgery was performed using the same methods, but the MCA was not coagulated.

### Oligonucleotides

AntimiRs targeting miR-126 and cRNA bound to α-tocopherol (Toc-cRNA) were synthesized by GeneDesign, Inc. (Osaka, Japan). The sequence information is shown in Supplementary Table S3 online. Cy3 fluorophores were covalently conjugated with the 5′ ends of antimiR. To generate Toc-HDO, equimolar amounts of antimiR and Toc-cRNA strand were heated in PBS (Nacalai tesque, Kyoto, Japan) at 95 °C for 5 min and cooled to 37 °C for 1 h.

### Cell culture and transfection

The bEnd3 cells (American Type Culture Collection, Manassas, USA) were cultured with high-glucose Dulbecco’s modified Eagle’s medium (DMEM; Wako, Osaka, Japan), supplemented with 10% fetal bovine serum (Life Technologies, Carlsbad, USA), 1 µg/ml streptomycin and 1 U/ml penicillin at 37 °C in a 5% CO_2_/95% air mixture. The cells were transfected with oligonucleotides by using Lipofectamine 2000 (Life Technologies, Carlsbad, USA), and then seeded in 24-well culture plates and incubated in DMEM for 6 h. The DMEM was replaced, and the cells were cultured for a total of 2 days before harvesting for analysis.

### Oxygen–glucose deprivation (OGD) procedures

To mimic ischemia in vitro, bEnd3 cells were subjected to OGD as described previously^46^. Briefly, bEnd3 cells in glucose-free DMEM (Wako, Osaka, Japan) were sealed and incubated in a closed chamber (Billups-Rothenberg Inc., Del Mar, USA) in 5% CO_2_/95% N_2_ for 2 h at 37 °C. The OGD was then terminated, and the cells were transfected with oligonucleotides by using Lipofectamine 2000.

### qRT-PCR analysis

PBS, antimiR #7, and Toc-HDO-antimiR #7 were administered to mice by tail vein injection. pMCAO model mice were injected at 3 h or an additional 7 days after the operation. Mice were deeply anesthetized and perfused with cold PBS through the left cardiac ventricle. Brain tissues from the ischemic region of the ipsilateral hemisphere and the contralateral cortex were dissected in the pMCAO model. Cerebral cortex was collected in sham-operated mice and normal mice. The brain tissues were immediately frozen in liquid nitrogen. Total RNA was isolated from brain tissues and bEnd3 cells by using a miRNeasy Mini Kit (QIAGEN, Hilden, Germany). cDNAs were synthesized using a Taqman MicroRNA Reverse Transcription Kit (Thermo Fisher Scientific Baltics UAB, Vilnius, Lithuania) and RT primer for miRNA examinations or Prime Script RT Master Mix (Takara, Kusatsu, Japan) for mRNA examinations. Subsequently quantitative PCR was performed using LightCycler 480 Probes Master (Roche, Mannheim, Germany) and a LightCycler 480 Real-Time PCR Instrument with pre-designed probes (Thermo Fisher Scientific, Waltham, USA, and Integrated DNA Technologies, Coralville, USA, see Supplementary Table S4 online). The qRT-PCR analyses were normalized to U6 small RNA for miRNA examinations or to *Gapdh* mRNA for mRNA examinations.

### Analysis of oligonucleotide concentrations in the brain tissues

pMCAO model mice were injected with Cy3-labeled antimiR #7 or Cy3-labeled Toc-HDO-antimiR #7 at 15 mg/kg at 3 h after the operation. Samples were collected from the ischemic region of the ipsilateral hemisphere and non-ischemic brain tissue of the contralateral hemisphere in pMCAO model mice at 1 h after intravenous administration, and then immediately frozen in liquid nitrogen. Brain tissues were homogenized in PBS, and then the signal intensity of Cy3 was measured by using an Infinite M1000 PRO microplate reader (Tecan, Männedrof, Switzerland).

### Immunohistochemical analyses

The brain tissues were fixed by transcardiac perfusion with 4% paraformaldehyde under deep anesthesia at 1 h after intravenous injection with Cy3-labeled antimiR #7 or Cy3-labeled Toc-HDO-antimiR #7 at 15 mg/kg. The brains were immersed in 4% paraformaldehyde overnight, and then sequentially immersed in distilled water, 7%, 15%, and 20% sucrose in PBS for a total of 2 days. The brains were frozen rapidly on dry ice and 20-μm thick sections were made with a cryostat (Leica Biosystems Nussloch GmbH, Nussloch, Germany). For detection of endothelial cells, the sections were immunolabeled with antibody against CD31 (1:50, Cat550274; BD Biosciences, Franklin Lakes, USA). Toc-HDO-antimiR #7 or antimiR #7 at 50 mg/kg were administered to the pMCAO model mice by two intravenous injections. After 14 days, the sections were immunolabeled with antibody against NeuN (1:200, MAB377; Sigma-Aldrich, St. Louis, USA) to visualize neuronal cells. Finally, the sections were incubated with fluorescence-labeled secondary antibodies and Hoechst stain, and were mounted with Vectorshield (Vector Laboratories, Burlingame, USA). All images were acquired using the software NIS-Element C installed in a laser scanning confocal microscopy (Nikon, Tokyo, Japan). The NeuN-positive cells were counted in regions of interest (ROIs) of 1 mm^2^ per section in the peri-infarct region. For PS staining, the sections were treated with 1% hydrogen peroxide for 30 min and blocked with 5% normal goat serum. Anti-PS antibody at a dilution of 1:8000 was applied to the sections for 2 days at 4 °C. Subsequently, the slides were incubated in secondary antibody (biotinylated anti-rabbit IgG antibody; Vector Laboratories, Burlingame, USA) at 1:1000 dilution for 2 h, followed by immunostaining with an Vectastain ABC kit (Vector Laboratories) for 1 h. The sections were visualized with diaminobenzidine and observed under a light microscope. Images was captured using the software cellSens standard installed in a light microscope (Olympus, Tokyo, Japan).

### Measurement of infarct volume

pMCAO model mice were intravenously injected at 3 h and 7 days post operation with PBS, antimiR #7, or Toc-HDO-antimiR #7 at 50 mg/kg, and brains were removed 14 days after pMCAO. To evaluate infarct volume, brain coronal sections were cut (20-µm thick) and stained with cresyl violet (Muto Pure Chemicals, Tokyo, Japan). The areas of the infarction were measured using public domain image analysis software (Image J, version 1.52i, National Institutes of Health, Bethesda, USA; http://imagej.nih.gov/ij). The infarct volume was calculated as a percentage of the contralateral hemisphere volume.

### Isolation of endothelial cells

The collected brain tissues were cut to pieces, and enzymatically degraded in 1 mg/ml collagenase type 2 (Worthington Biochemical Corporation, Lakewood, USA), 0.002 mg/ml DNase I (Sigma-Aldrich), and DMEM at 37 °C for 1 h. After centrifugation, the solution was replaced with 1 mg/ml collagenase/dispase (Roche, Mannheim, Germany), 0.002 mg/ml DNase I, and DMEM. The tissues were further incubated at 37 °C for 1 h. After centrifugation, the pellets were homogenized by pipetting in DMEM and the homogenates were filtered through a cell strainer. The resultant cell suspensions were centrifuged and discard the supernatant. Then, the pellets were suspended in the 20% BSA solution (containing 30% BSA [Sigma-Aldrich], 0.002 mg/ml DNase I, and DMEM) and centrifuged at 1000 g for 20 min at 4 °C. The pellets were suspended in purification buffer, containing 5% MACS BSA Stock Solution (Miltenyi Biotec, Bergisch Gladbach, Germany) in Auto MACS Rinsing Solution (Miltenyi Biotec). Then, the cell suspensions incubated on ice for 15 min with CD31 MicroBeads (Miltenyi Biotec). After centrifugation, the pellets were dissolved in purification buffer, and the suspension was poured onto an LS column (Miltenyi Biotec) and placed on a Quadro MACS Separator (Miltenyi Biotec). The LS columns were washed with purification buffer, leaving the BECs adsorbed on the columns. The LS columns were removed from the Quadro MACS Separator, and washed with purification buffer to obtain purified BECs.

### Microarray analysis

Microarray analysis was performed to produce mRNA expression profiles in (a) BECs from normal mice treated intravenously with Toc-HDO-antimiR #7 at 5 mg/kg, 15 mg/kg, or 50 mg/kg and (b) bEnd3 cells transfected with antimiR #7 at 30 nM. The BECs were isolated from the brain tissues by using the method described in the section above. mRNAs were extracted from the BECs and bEnd3 cells by using a miRNeasy Mini Kit. Total RNA (100 ng) extracted from the BECs and bEnd3 cells was converted to cDNA, in vitro transcription was performed, and Cy3-CTP was incorporated into the nascent cRNA by using the One-Color Microarray-Based Gene Expression Analysis/Low Input Quick Amp Labeling System (Agilent, Santa Clara, USA). After fragmentation, labeled cRNA was hybridized to Agilent SurePrint G3 Mouse GE 8 × 60 k Microarrays (Agilent, Santa Clara, USA) for 17 h at 65 °C. The microarray slides were then washed and scanned on an Agilent Microarray Scanner G2565BA (Agilent, Santa Clara, USA). The raw signals were log transformed and normalized by using the 75th percentile shift normalization method. The fold changes of gene expressions in each group were calculated relative to the control group.

### Luciferase reporter assay

We transfected bEnd3 cells with 50 ng of the MiCheck miRNA biosensor miR-126-3p, which carries the target sequences for miR-126-3p in the *Renilla* luciferase reporter gene, or psiCHECK-2 vector (Promega, Madison, USA) by using Lipofectamine 3000 (Life Technologies, Carlsbad, USA). After a 6-h incubation period, the transfection media was replaced by fresh medium. Then antimiRs were introduced by using Lipofectamine 2000. Luciferase reporter activities were measured by using the Dual-Glo Luciferase Assay System (Promega, Madison, USA) at 24 h after the last transfection step. *Renilla* luciferase activity was normalized to firefly luciferase activity.

### Evaluation of possible adverse effects

Mice were intravenously administered Toc-HDO-antimiR #7 at 5 mg/kg, 15 mg/kg, or 50 mg/kg. Blood samples were collected for serum biochemical analysis at 7 days after injection. Kidneys and livers were removed for histological analysis at 7 days after injection of Toc-HDO-antimiR #7 at 50 mg/kg. Kidney and liver tissues were fixed by transcardiac perfusion with 4% paraformaldehyde, and immersed in 4% paraformaldehyde overnight. The tissues were embedded in paraffin, and then were cut into 5-μm sections by using a microtome (ROM-308; Yamato Kohki Industrial Co, Tokyo, Japan). The sections were stained with hematoxylin and eosin (Muto Pure Chemicals).

### Statistical analysis

All data are presented as mean ± s.e.m. One-way analysis of variance (ANOVA) followed by a post hoc Tukey–Kramer test or Dunnett’s test was used to determine the statistical differences among various groups. *P* < 0.05 was considered to be statistically significant.

## Supplementary Information

Supplementary Information.

## Data Availability

The datasets generated and/or analyzed during the current study are available from the corresponding author on reasonable request.
