# Supplementary material for: Effective silencing of miR-126 after ischemic stroke by means of intravenous α-tocopherol–conjugated heteroduplex oligonucleotide in mice
Source: Sci Rep. 2021 Jul 9;11:14237. doi: 10.1038/s41598-021-93666-y (PMC8270953; doi:10.1038/s41598-021-93666-y)
Supplement: Supplementary file 1 — Supplementary Information. [file 41598_2021_93666_MOESM1_ESM.pdf]

Supplementary information

Effective silencing of miR-126 after ischemic stroke by means of intravenous α-tocopherol–conjugated heteroduplex oligonucleotide in mice

Motohiro Suzuki, Satoru Ishibashi, Eri Iwasawa, Takahiro Oguma, Yasuhiro Saito, Fuying Li, Shinichi Otsu, Keiko Ichinose, Kotaro Yoshioka, Tetsuya Nagata, and Takanori Yokota

Supplementary Fig. S1 Serum biochemical and histological analysis after injection of Toc-HDO-antimiR #7

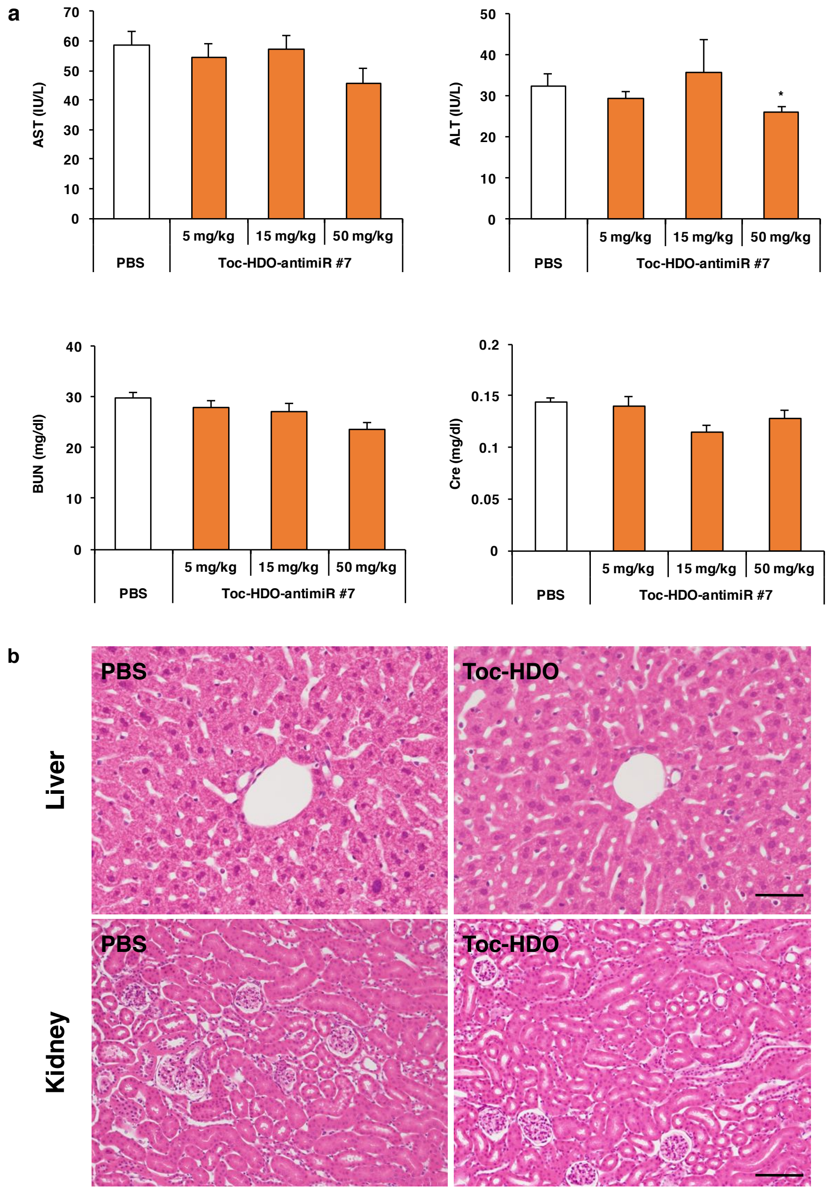

(a) Biochemical analysis of aspartate transaminase (AST), alanine transaminase (ALT), blood urea nitrogen (BUN), and creatinine (Cre) in blood serum from normal mice at 7 days following injection of Toc-HDO-antimiR #7 at 5 mg/kg, 15 mg/kg, or 50 mg/kg. Data are presented as the mean ± s.e.m. (n = 4 to 5 per group. **P* < 0.05 versus PBS control). *P* values were calculated using one-way ANOVA followed by Dunnett's test. (b) Hematoxylin and eosin staining of kidney and liver sections collected 7 days after the injection of Toc-HDO-antimiR #7 at 50 mg/kg. Upper scale bar, 50 μm; Lower scale bar, 100 μm.

Supplementary Fig. S2 Distribution of Cy3-labeled Toc-HDO-antimiR #7 in the ischemic region and contralateral cortex following pMCAO

a

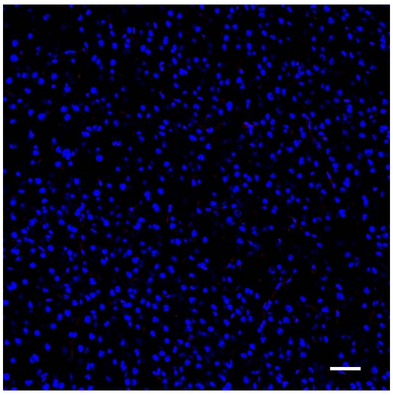

b

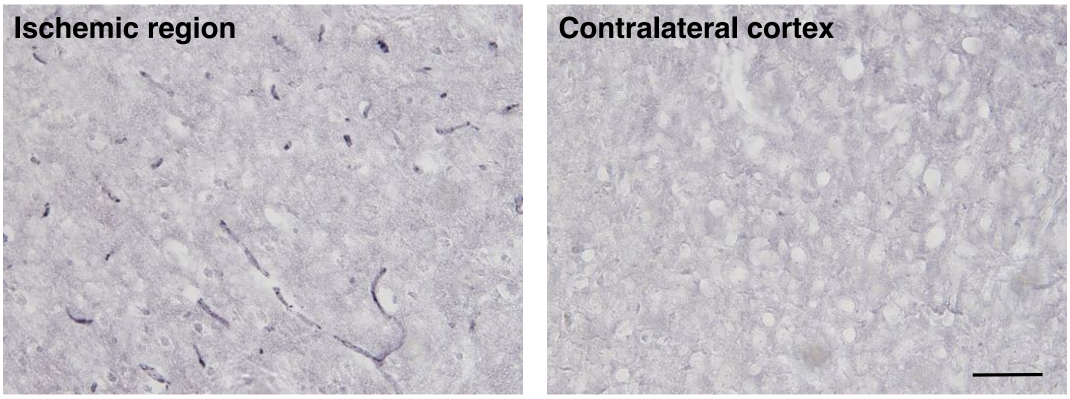

(a) Confocal laser scanning microscopic image of a section in the contralateral cortex after intravenous injection of Cy3-labeled Toc-HDO-antimiR #7 following pMCAO. Immunofluorescence images of Cy3-labeled Toc-HDO-antimiR #7 (red). Scale bars, 50 μm.

(b) Immunohistochemical staining with anti-phosphorothioate (PS) antibody in the ischemic region and contralateral cortex following intravenous administration of Cy3-labeled Toc-HDO-antimiR #7. Scale bar, 50 μm.

Supplementary Fig. S3 Expression levels of miR-126 and miR-126 downstream genes in bEnd3 cells transfected with antimiR scramble and Toc-HDO-antimiR scramble

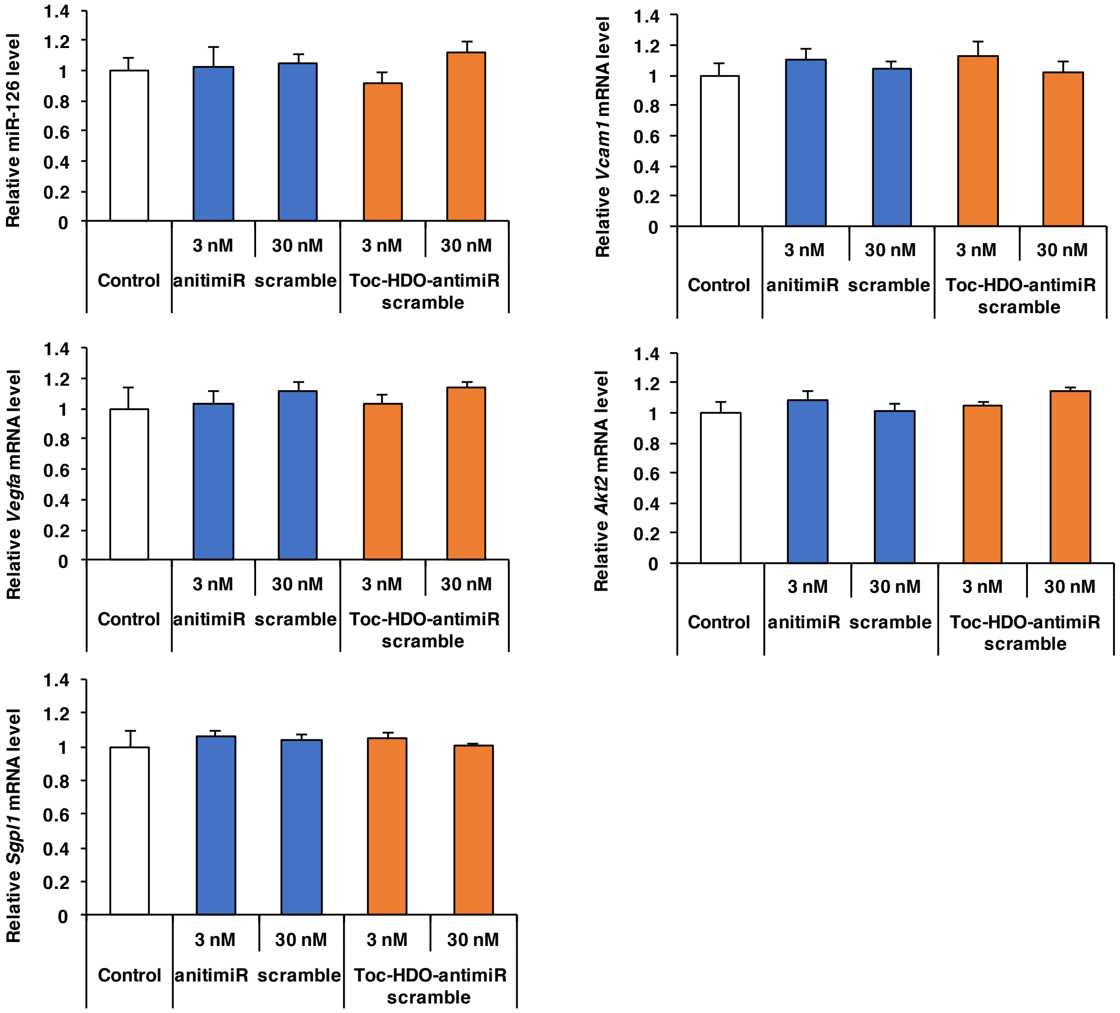

qRT-PCR analysis of miR-126 expression levels and *Vcam1, Vegfa*, *Akt*2, and *Sgpl1* mRNA levels in bEnd3 cells transfected with Toc-HDO-antimiR scramble or antimiR scramble at 3 nM or 30 nM. The expression levels are calculated relative to U6 small RNA levels for miR-126 expression experiments and glyceraldehyde-3-phosphate dehydrogenase (*Gapdh*) mRNA levels for mRNA experiments. Data are presented as the mean ± s.e.m. (n = 3 per group). When the groups were compared with their respective non-treated control, no results were statistically significant (all $P > 0.05$). *P* values were calculated using one-way ANOVA followed by Dunnett’s test.

Supplementary Fig. S4 Predicted binding sites between miR-126 and the identified target genes

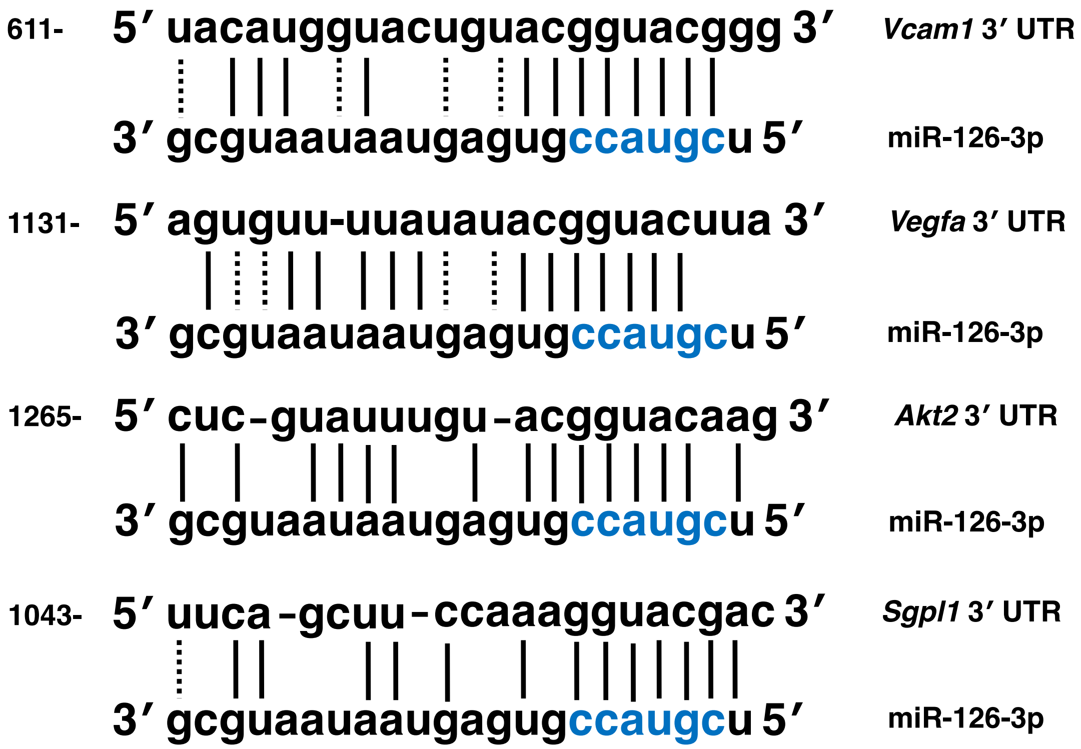

Sequence alignment of potential miR-126 binding sites in the 3′-UTR of *Vcam*1, *Vegfa*, *Akt2*, and *Sgpl1* genes. Blue, seed sequence.

Supplementary Fig. S5 Confocal microscopy analysis of neuronal damage and infarct volume after intravenous administration of Toc-HDO-antimiR #7

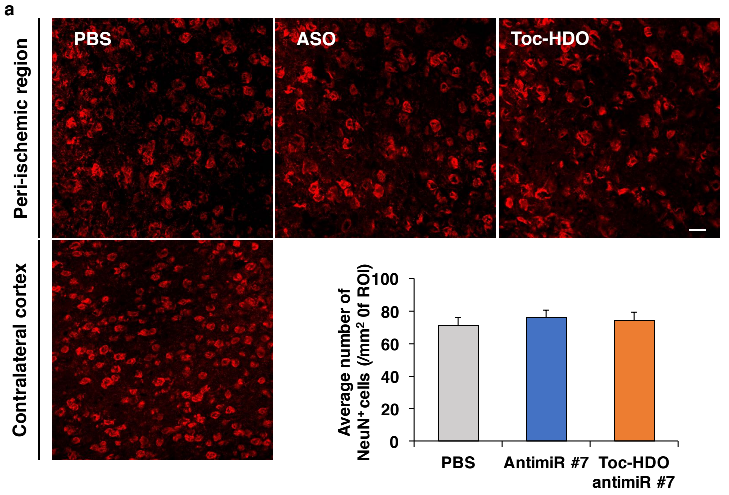

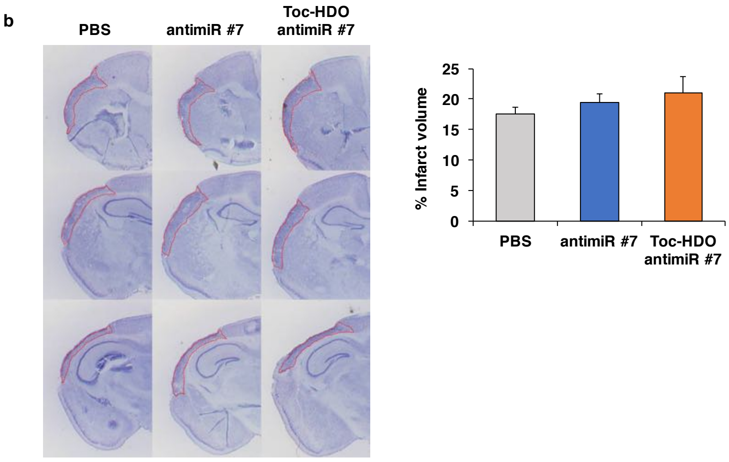

(a) Representative images show neurons stained with the neuronal marker NeuN (red) in the peri-infarct region or the contralateral cortex 14 days after pMCAO in the PBS group, antimiR #7 group, and Toc-HDO-antimiR #7 group. Scale bars, 25 µm. Bar graph showed the quantification of NeuN-positive neurons. Data are presented as mean ± s.e.m. (n = 3 per group). No significant differences among groups was found by one-way ANOVA followed by Tukey-Kramer test. (b) Cresyl violet staining of brain sections 14 days after pMCAO in the PBS group, antimiR #7 group, and Toc-HDO-antimiR #7 group. Infarct volume in the PBS group, antimiR #7 group, and Toc-HDO-antimiR #7 group is measured using Image J (version 1.52i; http://imagej.nih.gov/ij). Data are presented as mean ± s.e.m. (n = 8 to 9 per group). No significant differences among groups was found by one-way ANOVA followed by Tukey-Kramer test.

Supplementary Table S1. Histopathological findings in liver and kidney

Organ	Findings	PBS	Toc-HDO antimiR #7
Liver	Hepatocellular single cell necrosis, minimal	0	0
	Increased mitotic figures, minimal	0	0
Kidney	Tubular de/regeneration, minimal	0	0
	Tubular hyaline casts, minimal	0	0
Total number of animals		4	4

Liver and kidney sections stained with hematoxylin and eosin were analyzed under an optical microscope.

Supplementary Table S2. Identified miR-126 downstream genes by microarray analysis

	Fold change			
	Isolated brain endothelial cells			bEnd3 cells
mRNA	5 mg/kg	15 mg/kg	50 mg/kg	30 nM
Vcam1	1.30	1.47	1.56	2.83
Vegfa	1.22	1.2	1.7	5.23
Akt2	1.06	1.44	1.68	3.35
Sgpl1	1.39	1.57	1.59	2.52
Gbp2	1.44	1.81	2.09	5.49

Genes identified by microarray analysis in isolated BECs from normal mice after injection of Toc-HDO-antimiR #7 at 5 mg/kg, 15 mg/kg, or 50 mg/kg and in bEnd3 cells transfected with antimiR 30 nM. Fold changes are calculated relative to control.

Supplementary Table S3. Oligonucleotide sequences

Name	Sequence
antimiR #1	c*g*c*a*t*t*a*t*t*a*c*t*c*a*c
antimiR #2	g*c*a*t*t*a*t*t*a*c*t*c*a*c*g
antimiR #3	c*a*t*t*a*t*t*a*c*t*c*a*c*g*g
antimiR #4	a*t*t*a*t*t*a*c*t*c*a*c*g*g*t
antimiR #5	t*t*a*t*t*a*c*t*c*a*c*g*g*t*a
antimiR #6	t*a*t*t*a*c*t*c*a*c*g*g*t*a*c
antimiR #7	a*t*t*a*c*t*c*a*c*g*g*t*a*c*g
antimiR #8	t*t*a*c*t*c*a*c*g*g*t*a*c*g*a
cRNA	C*G*U*ACCGUGAGU*A*A*U
antimiR-scramble	t*c*c*a*t*g*c*a*g*g*a*t*c*t*a
cRNA-scramble	A*G*G*UACGUCCUA*G*A*U

Lower case letters represent DNA; lower case underlined letters represent LNA;
c denotes LNA 5-methylcytosine
Upper case letters represent RNA; asterisks represent phosphorothioate internucleotide linkage.

Supplementary Table S4. Primer information

Gene name	Product name	Assay ID or Catalog #
hsa-miR-126-3p	TaqMan MicroRNA Assays	002228
U6 snRNA	TaqMan MicroRNA Assays	001973
Vcam1	PrimeTime Predesigned qPCR Assays	Mm.PT.58.9687546
Akt2	PrimeTime Predesigned qPCR Assays	Mm.PT.58.8836469
Vegfa	PrimeTime Predesigned qPCR Assays	Mm.PT.58.31754187
Sgpl1	TaqMan Gene Expression Assays	Mm00486079_m1
Gapdh	Mouse GAPD (GAPDH) Endogenous Control	4352932E
